# An APETALA2/ethylene responsive factor transcription factor GmCRF4a regulates plant height and auxin biosynthesis in soybean

**DOI:** 10.3389/fpls.2022.983650

**Published:** 2022-09-06

**Authors:** Zhiyong Xu, Ruikai Wang, Keke Kong, Naheeda Begum, Aisha Almakas, Jun Liu, Hongyu Li, Bin Liu, Tuanjie Zhao, Tao Zhao

**Affiliations:** ^1^National Center for Soybean Improvement, Key Laboratory of Biology and Genetics and Breeding for Soybean, Ministry of Agriculture and Rural Affairs, State Key Laboratory of Crop Genetics and Germplasm Enhancement, Nanjing Agricultural University, Nanjing, China; ^2^The National Key Facility for Crop Gene Resources and Genetic Improvement (NFCRI), Institute of Crop Science, Chinese Academy of Agricultural Sciences, Beijing, China

**Keywords:** soybean, plant height, auxin, cytokinin response factor, GmCRF4a

## Abstract

Plant height is one of the key agronomic traits affecting soybean yield. The cytokinin response factors (CRFs), as a branch of the APETALA2/ethylene responsive factor (AP2/ERF) super gene family, have been reported to play important roles in regulating plant growth and development. However, their functions in soybean remain unknown. This study characterized a soybean *CRF* gene named *GmCRF4a* by comparing the performance of the homozygous *Gmcrf4a-1* mutant, *GmCRF4a* overexpression (*OX*) and co-silencing (*CS*) lines. Phenotypic analysis showed that overexpression of *GmCRF4a* resulted in taller hypocotyls and epicotyls, more main stem nodes, and higher plant height. While down-regulation of *GmCRF4a* conferred shorter hypocotyls and epicotyls, as well as a reduction in plant height. The histological analysis results demonstrated that GmCRF4a promotes epicotyl elongation primarily by increasing cell length. Furthermore, GmCRF4a is required for the expression of *GmYUCs* genes to elevate endogenous auxin levels, which may subsequently enhance stem elongation. Taken together, these observations describe a novel regulatory mechanism in soybean, and provide the basis for elucidating the function of GmCRF4a in auxin biosynthesis pathway and plant heigh regulation in plants.

## Introduction

Soybean [*Glycine max* (L.) Merr.] is an economically important crop cultivated globally, providing a valuable source of cooking oil and plant protein for human consumption (Lee et al., 2011). Although, the global soybean production has gradually increased, the yield per unit of soybean is relatively low compared to other cereal crops including wheat, maize, and rice, which have achieved great success in developing ideal plant types (Liu et al., 2020; Vogel et al., 2021). It has been noted that genetic dissection and the use of specific plant-type traits, including plant height, can improve the efficiency of soybean high-yield breeding (Liu et al., 2020). Plant height, as a complex quantitative trait, is governed by abundant genes and a variety of hormones, such as gibberellin, auxin, and cytokinin (Wang et al., 2017, 2021; Zhang et al., 2017; Dong et al., 2019; Liao et al., 2019; Castorina and Consonni, 2020; Bai et al., 2021). During the last century’s “Green Revolution,” the *Rht1* (*Reduced height 1*) gene of wheat (Rockwell and Hongay, 2019), the *Sd1* (*Semi-dwarf 1*) gene of rice (Ren et al., 2019), and the *D8* (*Dwarf-8*) gene of maize (Huang et al., 2019) all regulated plant height by adjusting gibberellic acid (GA) contents. In addition to GA pathway, genes affecting plant height *via* the auxin pathway have also been identified in several crops. For example, overexpression of the *ZmPIN1a* (*PIN-FORMED1a*) gene in maize reduces plant height, internode length, and ear height (Li et al., 2018a). Knocking out the members of the *TRANSPORT INHIBITOR RESISTANT1/AUXIN SIGNALING F-box* (*OsTIR1/AFB*) gene family alters plant height and yield in rice (Guo et al., 2021). Growth habit is an agronomically important trait associated with the domestication in soybean. According to the termination time of apical stem growth, most soybeans can be divided into two growth habits: indeterminate and determinate, which brings about changes in plant height and yield. Subsequently, the key genes *Determinate 1* (*Dt1*) and *Dt2* genes regulating plant height and growth habits of soybean were found (Tian et al., 2010; Ping et al., 2014). Deletion of *Dwarf Mutant 1* (*GmDW1*) (Li et al., 2018b), *GmAP1s* (*Apetala1*) (Chen et al., 2020), and *GmLHY* (*Late elongated hypocotyl*) (Cheng et al., 2019) genes in soybean can also regulate plant height by changing endogenous GA content. Overexpression of both an APETALA2-like gene *GmTOE4a* and an ABI3/VP1 gene *GmRAV* can cause reduced plant height and internode length with lower GA levels in soybean plants (Zhao et al., 2015; Xue et al., 2022). Nevertheless, few auxin related genes have been reported to affect plant height in soybean.

The AP2/ERF superfamily, as one of the largest transcription factor families in plants, can be divided into five subfamilies: APETALA2 (AP2), RAV (ABI3/VP1), Dehydration Responsive Element Binding Protein, ethylene responsive factor (ERF) and Soloist according to the number and binding sequence of AP2/ERF domains (Nakano et al., 2006; Wang, 2019). The AP2/ERF family genes play multiple roles in regulating plant defense responses to stress as well as growth and development (Licausi et al., 2013; Chen et al., 2015; Gan et al., 2019; Ma et al., 2020; Zhang et al., 2020; Zhao et al., 2020). For example, the cytokinin response factors (CRFs) proteins, as a branch of the ERF subfamily, play essential roles in plant growth and development, nitrogen uptake, biological and abiotic stress, and cytokinin and auxin response (Cutcliffe et al., 2011; Jeon et al., 2016; Kim, 2016; Zwack et al., 2016; Hallmark and Rashotte, 2019; Zong et al., 2021). In Arabidopsis, there are 12 *CRF* genes, with *CRF4* being one of the few whose transcription is not induced by cytokinin (Rashotte et al., 2006; Cutcliffe et al., 2011; Zwack et al., 2012). The *crf4* mutant was initially found to be sensitive to low temperature treatment (Zwack et al., 2016). While under normal conditions, *crf4* mutants and *CRF4* overexpression lines (*CRF4OX*) showed no obvious phenotypic alterations, except for a slight increase in primary root length of *CRF4OX*. In addition, under low nitrogen conditions, the *CRF4* overexpression significantly reduced ^15^NO_3_^−^ uptake and plant biomass, including reduced dry weight, primary root length, and lateral root number (Varala et al., 2018).

In this study, we tested the function of *GmCRF4a* gene in more detail by characterizing its expression patterns, subcellular localization and by *GmCRF4a* mutant, overexpressing, and co-silencing lines to dissect the roles of this transcription factor in regulation of plant height. We observed that the plant height of *Gmcrf4a-1* mutant and co-silencing lines were significantly decreased than that of the WT. We also observed the relative expression levels of auxin biosynthesis genes and the auxin content in the *Gmcrf4a-1* mutant and co-silencing lines were significantly lower than in WT. These results suggested that *GmCRF4a* may directly regulate plant height by mediating key components of the auxin pathway. Moreover, the phenotype of *GmCRF4a* overexpression lines further confirmed the above results. Our findings suggest that the manipulation of *GmCRF4a* gene should facilitate improvement in plant height in soybean.

## Materials and methods

### Plant materials and growth conditions

The soybean cultivar Tianlong 1 (TL1) is an elite material for genetic transformation. For the seedling phenotypes, the wild type (TL1), *GmCRF4a* knockout mutants (*Gmcrf4a-1*), *GmCRF4a* co-silencing line (*CS*), and *GmCRF4a* overexpression lines (*OX1* and *OX2*) were grown under 16 h light/8 h dark conditions or 12 h light/12 h dark conditions with 250–300 μmol·m^−2^ s^−1^ light at 25°C for 11 days. For the field phenotypes, the materials were grown under natural conditions (about 14.5 h-light conditions) in a field in Beijing (40.1° N, 116.7° E) with two plot replications. The seeds were planted in a 3.0 m row, with 0.6 m separating rows and a space of 0.3 m between adjacent plants. The plant height and node number of main stem were investigated and photographed during the period of co-silencing line (*CS*) R8, and the growth period and grain status of each material were counted after harvest.

### Phylogenetic analysis AP2/ERF genes

The peptide sequences of AP2/ERF in soybean and Arabidopsis thaliana were selected with the Pfam name PF00847 (ap2 domain) in a threshold e-value < 10^−5^ from Phytozome v13.0.^1^ All the soybean AP2/ERF protein sequences were aligned with Multiple Sequence Alignment v7.273 (Katoh and Standley, 2013), and then back-translated into coding sequence by ParaAT (Zhang et al., 2012). At last, Maximum-Likelihood (ML) phylogenetic tree was generated using Fasttree for a larger number of sequences (Price et al., 2009). The detailed list is presented in Supplementary Table S1. For the *CRF4* phylogenetic tree the *GmCRF4a* homologous genes in *Arabidopsis*, soybean, maize, and rice were used to constructed Maximum Likelihood tree by MEGA 7.0 software. For the protein sequence alignment and critical domain analysis were conducted by DNAMAN software.

### Expression patterns of GmCRF4a

Tissue-specific expression patterns data came from the soybean Expression Atlas.^2^ The median TPM per tissue-stir condition was selected, and the graphic was created by GraphPad Prism 9 software. Tissue-specific expression data is given in Supplementary Table S2.

### Subcellular localization

The full-length cDNA of *GmCRF4a* was cloned into the *pA7-YFP* vector. *GmCRF4a-YFP* was transiently expressed in soybean mesophyll protoplasts as described in our previous work (Xiong et al., 2019). The *pA7-GmMYB29-RFP* plasmid was used as a nuclear marker (Chu et al., 2017). The fluorescence signal was observed under a confocal microscope (Zeiss LSM700) after 16 h of transformation at room temperature in the dark.

### Vector construction and soybean transformation

To generate the overexpression vectors, the full-length *GmCRF4a* was cloned into the Gateway entry vector pDONRZeo, and then fused into the destination binary vector *pEarleyGate-101-eYFP* vector using the Gateway recombination system (Invitrogen). To generate the CRISPR-Cas9 knockout soybean mutants, gRNAs were designed using the web tool CRISPR-P,^3^ and constructed into the *p0645* vector. According to the previously described method (Lyu et al., 2021), the Agrobacterium-cotyledonary node transformation system was used to generate overexpression lines and gene editing mutants. To screen for homozygous mutants, transgenic lines were propagated by self-pollination for at least two generations. Homozygous mutations were identified by DNA sequencing (Supplementary Figure S4A). For CS lines, it was obtained by screening for co-suppression lines with the opposite phenotype to the overexpressed lines. GmCRF4a expression was detected by qPCR experiments.

### Histological analysis

For epicotyl longitudinal sections, seedlings were grown under 12 h light/12 h dark with 250–300 μmol·m^−2^ s^−1^ light at 25°C for 11 days and then stained with safranin-O-fast green by paraffin sections (De Micco and Aronne, 2007). The images were observed using a ZEISS Imager M2 (Jena, Germany) after staining. The cell size was calculated by ImageJ software.

### Hormone determination

The seedlings were grown under 12 h light/12 h dark conditions with 250–300 μmol·m^−2^ s^−1^ light at 25°C for 11 days. For auxin concentration measurement, five single plants’ epicotyls were measured. The IAA enzyme-linked immunoassay (ELISA) kit (Meimian, MM-0953O1, Yancheng, China) was used for the determination of auxin. Please see kit description for details.

### Gene expression analysis

Total RNA was extracted from 13-day old soybean leaves under 12 h light/12 h dark conditions using Trizol (Invitrogen, 15,596–026, California, United States). Quantitative real-time polymerase chain reaction (qRT-PCR) was performed in 384-well optical plates on the QuantStudio 7 Flex (Massachusetts, United States) using SYBR Green RT-PCR kit (Vazyme, Q221-01, Nanjing, China). The *GmActin11* was used as an internal control. Three independent biological replicates and two mechanical replicates were evaluated. Related genes and primer lists are presented in Supplementary Table S3.

### Statistical analysis

Two-sided *t*-test and ANOVA were performed on the data using GraphPad Prism 9 software.

## Results

### Expression profile and subcellular localization of GmCRF4a

**Figure 1:**
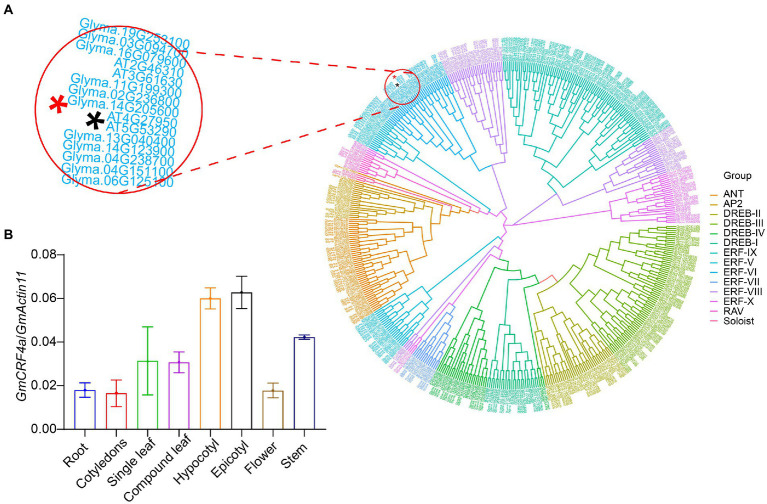
A phylogenetic tree and tissue-specific analysis of **(A)** homology analysis of *AP2/ERF* genes in soybean and *Arabidopsis thaliana*. These include 354 soybean genes and 146 *Arabidopsis thaliana* genes. Different colors are marked according to different subfamilies, where ANT represents AINTEGUMENTA. The black asterisk represents *CRF4* in *Arabidopsis thaliana*; the red asterisk represents *GmCRF4a* in soybean. And the upper left corner is a partial enlargement. **(B)** Transcriptional levels of *GmCRF4a* in various tissues of TL1*. GmActin11* as an internal control. Data are displayed as means ± s.d. (*n* = 3).

In our previous research we identified an *AP2/ERF* family gene *GmCRF4a (Glyma.14G205600)*, which originally discovered as one of the fine-mapping candidate genes and turned out to be a non-target gene, involved in the regulation of important traits such as plant height and flowering in soybean. Therefore, we conducted further studies on this gene. *GmCRF4a* encodes a protein of 282 amino acid residues with a predicted molecular mass of 31.7 kDa. BLASP search of the *Arabidopsis* database revealed that *GmCRF4a* shares the highest identity with *AT4G27950*, *Cytokinin Response Factor 4 (CRF4)*, which led us to designate *Glyma.14G205600* as *GmCRF4a*, the first identified in *Arabidopsis CRF4* homologue. Protein analysis of the deduced amino acid sequence revealed that *GmCRF4a* contains a typical AP2/ ERF domain. Further amino acid alignment analysis showed that GmCRF4a shares high similarity at AP2/ERF domain with the homologues in soybean (*Glyma*.*04G238700*, *Glyma*.13G040400, *Glyma*.*02G236800*, *Glyma*.*19G253100*, *Glyma*.*03G255500*, *Glyma*.*03G094700*, *Glyma*.*20G215700*, *Glyma*.*05G214400*, *Glyma*.*11G199300*, *Glyma*.*11G019000*, *Glyma*.*14G205600*, *Glyma*.*14G123900*, *Glyma*.*08G020900*, *Glyma*.*01G224100*, *Glyma*.*06G125100* and *Glyma*.*16G079600*), maize (*Zm00001d034605*, *Zm00001d036251*, *Zm00001d044004*, *Zm00001d039324*, *Zm00001d008968*, *Zm00001d011499*, *Zm00001d045262*), rice (*LOC*_*Os01g46870*, *LOC*_*Os01g12440*, *LOC*_*Os06g06540*) and *Arabidopsis* (*CRF1*, *CRF2*, *CRF3*, *CRF4*, *CRF5* and *CRF6*), (Supplementary Figure S1). Phylogenetic analysis showed that *GmCRF4a* shares 50–85% high identity with soybean homologues, and about 41% identity with the Arabidopsis genes (Supplementary Figure S2). Homologous genes in rice and maize clustered together independently, with lower homology to GmCRF4a. To determine subgroup of *GmCRF4a* in *AP2/ERF* gene family, 354 soybean *AP2/ERF* genes from cultivated soybean Williams 82 and 146 Arabidopsis *AP2/ERF* genes were selected for homology comparison, and the phylogenetic tree was constructed by referring to previous *AP2* family classification methods (Wang, 2019). Cluster analysis revealed that the gene *GmCRF4a* (Figure 1A; Red Asterisk) belonged to the *ERF-VI* subfamily of *AP2/ERF.*

**Figure 2:**
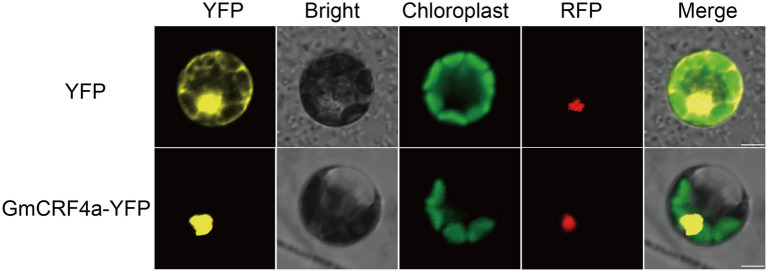
Subcellular localization of GmCRF4a-YFP fusion protein in soybean mesophyll protoplasts. The GmCRF4a-YFP fusion proteins were located in the nucleus. YFP was used as a control. The GmMYB29-RFP fusion protein was used as a nuclear marker Scale bars = 5 μm.

To get insight into the function of the *GmCRF4a* gene, we detected the expression level of *GmCRF4a* in different tissues under the background of TL1. The results showed that the *GmCRF4a* gene was evenly expressed in all tissues (Figure 1B), suggesting that *GmCRF4a* may be function as a transcriptional factor to regulate soybean growth and development of entire life cycle. Then we compared the *GmCRF4a* gene expression level among different tissues using the data accessed from the soybean Expression Atlas.^4^ The results were similar to the data provided by the qPCR, that is, *GmCRF4a* was expressed in all tissues (Supplementary Figure S3). To determine the subcellular localization of GmCRF4a, we transiently expressed the GmCRF4a-YFP fusion protein in soybean mesophyll protoplasts. The fluorescent images demonstrated that GmCRF4a-YFP was colocalized with nuclear markers (Figure 2), while the control protein YFP was only detectable in the intracellular region.

### GmCRF4a increases plant height

**Figure 3:**
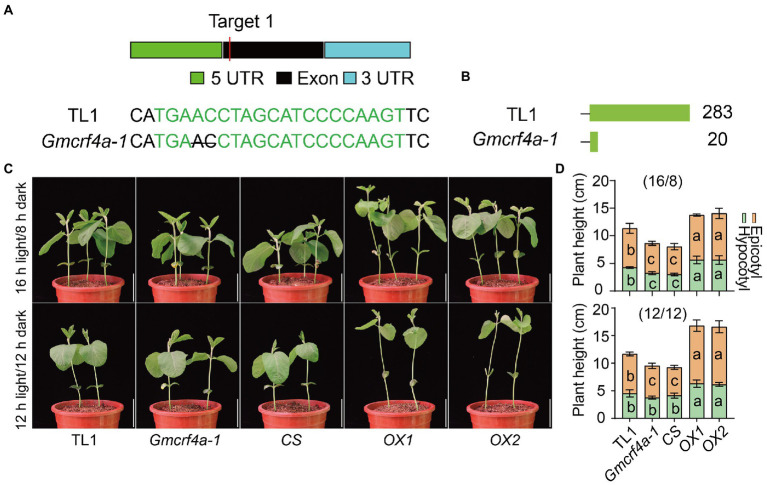
The phenotypes of wild type (TL1), *GmCRF4a* knockout mutants (*Gmcrf4a-1*), *GmCRF4a* co-silencing line (*CS*), and *GmCRF4a* overexpression lines (*OX1* and *OX2*) were compared at the seedling stage. **(A)** The sgRNA designed to position and base change *Gmcrf4a-1*, the black line within the target site, denotes nucleotide deletion. **(B)** Schematic diagram comparing intact and mutated GmCRF4a proteins. The number of amino acids behind each protein. **(C)** Plants height of wild type (TL1), *GmCRF4a* knockout mutant (*Gmcrf4a-1*), *GmCRF4a* co-silencing line (*CS*), and *GmCRF4a* overexpression lines (*OX1* and *OX2*) at 11 days under 16 h light/8 h dark or 12 h light/12 h dark conditions. Plant height statistics **(D)**: data from C. The letters in the yellow areas indicate significant differences between epicotyls, and the letters in the green areas indicate significant differences between hypocotyls (*p* < 0.05). It was determined by one-way ANOVA (Tukey’s multiple comparisons test). Data are means ± s.d. (*n* > 4).

To dissect the role of GmCRF4a in soybean development, we knocked out the *GmCRF4a* gene by CRISPR/Cas9 genome editing technology. We generated a homozygous mutant line, *Gmcrf4a-1,* which carries two base deletions (ΔAC) in the exon resulting in premature termination of protein translation (Figures 3A,B; Supplementary Figure S4A). Furthermore, we made the *35S:GmCRF4a-YFP* construct and obtained two overexpression lines (*OX1* and *OX2*) and one co-silencing line (*CS*) using the Agrobacterium-cotyledonary node transformation system (Figure 3C; Supplementary Figure S4B). Subsequently, these lines were planted under 16 h light/8 h dark growth conditions and 12 light/12 dark growth conditions for phenotypic analysis. The results showed that, compared with the wild-type TL1, the *OX1* and *OX2* lines showed an increased plant height phenotype, with longer hypocotyls and epicotyls, under both 16 h light/8 h dark growth conditions or 12 light/12 dark growth conditions (Figures 3C,D). In sharp contrast, the plant height of the *Gmcrf4a-1* mutant and *CS* line were significantly decreased in comparison to that of TL1 (Figures 3C,D). Under natural field conditions, *OX1* line produced more main stem nodes (MSNs) and was taller than TL1, while the *CS* line was dwarf with less MSN (Supplementary Figures S5A–C). In addition, the growth period of the *OX1* line was prolonged, while that of the *CS* line was shortened in field conditions (Supplementary Figure S5D). Notably, the overall phenotypes of the *CS* line are relatively stronger than that of the *Gmcrf4a-1* mutant. This is possibly because not only the *GmCRF4a* gene but also its homologous genes *Glyma.02G236800* and *Glyma.14G123900* were co-silenced in the *CS* line (Supplementary Figures S6A,B). Taken together, these results suggested that the *GmCRF4a* gene positively regulates plant height by increasing stem elongation, node number, and growth period.

### GmCRF4a promotes cell elongation

**Figure 4:**
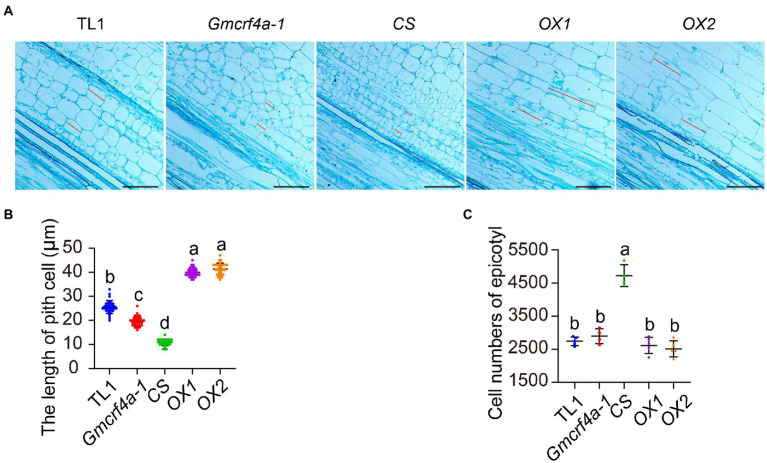
The organization structure and statistical analysis of epicotyls in five individuals. **(A)** Structure of pith cells in the epicotyls of 5 individuals at 11 days under 12 light/12 dark conditions. The cells between these two red lines represent the measurement part, scale bars = 30 μm. **(B)** The length of single pith cell in five individuals, data from A, each material measured 30 cells and 3 biological replicates. **(C)** Cell numbers of epicotyls in five individuals, data from the average length of epicotyl/the average of single cell length. Lowercase letters indicate significant differences (*p* < 0.05).

To investigate how *GmCRF4* regulates stem elongation at cellular level, we used the epicotyls of 11-day-old plants grown under 12 light/12 dark conditions for longitudinal section (Figure 4A). The histological results showed that the pith cell length of the *OX* lines increased, whereas that of the *Gmcrf4a-1* mutant and *CS line* decreased in comparison to the wild-type TL1 (Figure 4A). The statistical results demonstrated the pith cell length of the *OX1* and *OX2* lines increased by 60 and 70%, the mutant *Gmcrf4a-1* decreased by 20%, and the *CS* line decreased by 60%, respectively (Figure 4B). The number of pith cells in each line was estimated by the ratio of the epicotyl mean length to the pith cell mean length. The results showed that, although the number of pith cells in the *CS* line increased, the cell number of the *OX* lines and *Gmcrf4a-1* mutant did not change significantly in comparison to TL1 (Figure 4C). These results suggested that *GmCRF4a* promoted epicotyl elongation mainly by increasing cell length.

### GmCRF4a enhances auxin biosynthesis

**Figure 5:**
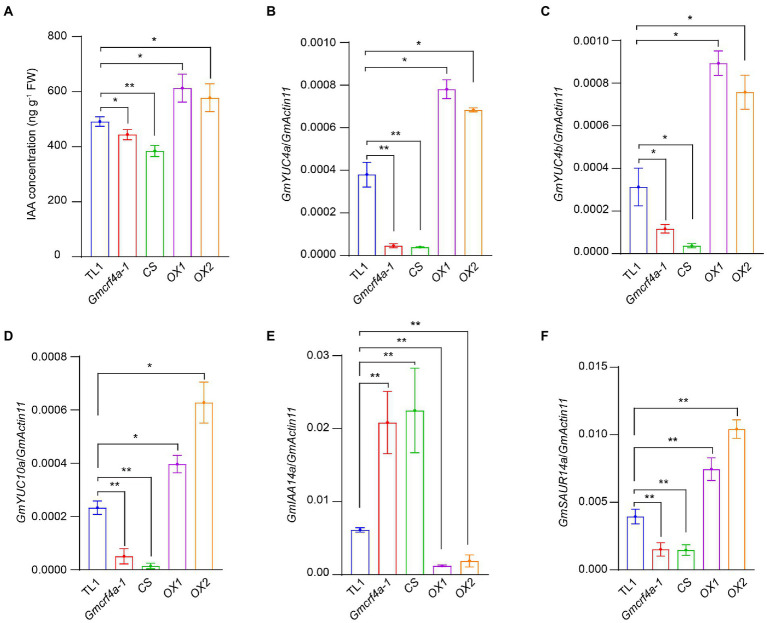
IAA concentration and IAA related gene expression levels in five individuals. **(A)** The concentration of IAA in five individuals for 11 days under SD. **(B–F)** Expression levels of IAA-related genes *GmYUC4a* **(B)**, *GmYUC4b* **(C)**, *GmYUC10a* **(D)**, *GmSAUR14a* **(E)** and *GmIAA14a* **(F)** in the indicated lines. *GmActin11* as an internal control. The asterisk indicates a statistically significant difference from the wild type by a two-sided *t*-test (^*^p < 0.05, ^**^p < 0.01). Data are displayed as means ± s.d. (*n* = 3).

To test if *GmCRF4a* enhances stem elongation through the auxin pathway, we analyzed the endogenous 3-indoleacetic acid (IAA) content in the epicotyl of 11-day-old plants grown under 12 light/12 dark conditions. The results indicated that the total IAA content substantially declined in the *Gmcrf4a-1* mutant and *CS* line, but significantly increased in the *OX1* and *OX2* lines, respectively (Figure 5A). Next, we test the expression of *YUCs* (*YUCA*) genes which play a key role in the auxin synthesis pathway (Chen et al., 2014; Chao et al., 2020). The qRT-PCR experiments were performed using the indicated lines. The results revealed that auxin synthesis genes *GmYUC4a*, *GmYUC4b*, and *GmYUC10a* were down-regulated in the *Gmcrf4a-1* mutant and *CS* line, but up-regulated in the *OX1* and *OX2* lines (Figures 5B–D). To be noted, the expressions of auxin transport carriers *GmPIN1a* and *GmPIN1b* were not changed significantly in the *GmCRF4a* mutant and overexpression lines (Supplementary Figure S7). These results indicated that *GmCRF4a* may increase auxin content by up-regulating the expression of *GmYUC* genes.

We further tested the expression of *AUX/IAA* (*Auxin/indole-3-acetic acid*) genes, the negative regulator of auxin signal transduction pathway (Sauer et al., 2006; Trenner et al., 2017; Xu et al., 2019). The results demonstrated that the *GmIAA14a* gene, a soybean *IAA14* member, was up-regulated in the *Gmcrf4a-1* mutant and *CS* line, but down-regulated in the *OX1* and *OX2* lines (Figure 5E). In addition, a soybean auxin-responsive gene *GmSAUR14a* (*Small auxin up RNA*) was down-regulated in the *Gmcrf4a-1* mutant and *CS* line, but up-regulated in the *OX1* and *OX2* lines (Figure 5F). Taking together, the above results reinforce the hypothesis that *GmCRF4a* enhances auxin content and further regulates plant height by promoting auxin synthesis gene expression.

## Discussion

In the past few decades, the green revolution has promoted the significant yield increase of gramineous crops such as rice and wheat resulting from the development of semi-dwarf plant architecture. However, it has been difficult to be applied in dicotyledonous crops such as soybeans. Actually, reducing plant height often leads to the reduction of biomass and nodes number, which directly affects the ultimate yield of soybean (Liu et al., 2020). The ideal architecture for soybeans could include strong stems, shorter internode lengths, and higher node numbers. To achieve this goal, the identification and functional characterization of genes that contribute to specific aspects of soybean architecture, such as the plant height of soybeans, will be critical to fully exploiting soybean genomes for crop improvement. In this study, we characterized a soybean CRF gene *GmCRF4a*, and function analysis results suggested *GmCRF4a* may positively regulate the plant height through affecting auxin pathway.

*CRF* genes belong to a subset of AP2/ERF transcription factors superfamily and originally function as CRFs in *Arabidopsis*, with one exception that *CRF4* does not transcriptionally respond to the cytokinin treatment (Rashotte et al., 2006; Cutcliffe et al., 2011; Zwack et al., 2012). Our amino acid alignment and phylogenetic analysis showed that the *GmCRF4a* gene had the highest identity to the *CRF4* gene in Arabidopsis. Supplementary Figures S1, S2, suggesting that GmCRF4a maybe have a similar function to CRF4. Further expression pattern analysis results indicated the soybean *GmCRF4a* was ubiquitously expressed in all detected tissues, especially highly expressed in hypocotyl, embryo, and suspensor (Figure 1B). In contrast, *Arabidopsis CRF4* is also widely expressed in the vascular system of plant root tip, stem, leaf, and flower, but not in stem apex (Zwack et al., 2012). In addition, the homologues of *Arabidopsis CRF4* in other species also exhibit various expression patterns. For example, tomato *SlCRF4* is also expressed in roots, stems, leaves, and flowers, but more in roots (Shi et al., 2012). Chinese cabbage *BrCRF4* is highly expressed in stamens, while *BolCRF4c* in *Brassica oleracea* and *BnaCRF4e* in *Brassica napus* are preferentially expressed in pistils (Liu et al., 2013; Kong et al., 2018). *PtERF85*, the homologous gene of *CRF4* in aspen trees, is highly expressed in phloem and vascular cambium, the highest in xylem expansion area and less in mature xylem (Seyfferth et al., 2021). The significant differences of expression pattern among the homologues of *CRF4* might imply their functional differences in different species.

In *Arabidopsis*, CRFs play an important role in embryo development, morphological structure, biological and abiotic stress. For example, *crf2*, *crf3*, *crf5*, *crf3crf6*, *crf2,5,6* and *crf1,3,5,6* single and multiple mutants led to various morphological changes in hypocotyls, main and lateral roots, leaf size, and leaf senescence (Rashotte et al., 2006; Jeon et al., 2016; Kim, 2016; Raines et al., 2016; Hallmark and Rashotte, 2019; Wang et al., 2020; Park et al., 2021). However, the altered *CRF4* expression did not exhibit visible phenotypic changes, except that a slight increase in primary root length of *CRF4OX*, and it responded to low temperature and low nitrogen levels (Zwack et al., 2016; Varala et al., 2018). Inconsistent with function of *Arabidopsis CRF4*, altering expression of *GmCRF4a* in soybean resulted in a variety of phenotypic changes. Knock out *GmCRF4a* led to the decreased pith cell length, lower hypocotyls and epicotyls, fewer MSNs, lower plant height and a shorter growth period, while *GmCRF4a* overexpressing plants exhibited opposite phenotype (Figure 3; Supplementary Figure S5), indicating a significant functional difference of *GmCRF4a* with its *Arabidopsis* homologue *CRF4*. In addition, it is reported that *Arabidopsis CRF4* homologous genes in other species are related to abiotic stress response. Under salt treatment, the expression of *SlCRF4* in tomato, *BnaCRF4b* and *BnaCRF4e* in *Brassica napus* were up-regulated, while the expression of *BolCRF4b* in *Brassica oleracea* and *BniCRF4c* in *Brassica nigra* were down regulated (Kong et al., 2018). After ethylene and salicylic acid treatment, the expression of *SlCRF4* increased significantly, but there was no significant change under jasmonic acid treatment (Shi et al., 2012). After ABA treatment, the expression of *BrCRF4*, *BolCRF4b*, *BniCRF4a*, *BniCRF4b* and *BniCRF4c* increased (Liu et al., 2013; Kong et al., 2018). After 6-BA treatment, the expression of *BnaCRF4e* was up-regulated, while the expression of *BolCRF4a*，*BolCRF4b*，*BniCRF4a*，*BniCRF4b* and *BnaCRF4c* were down-regulated (Kong et al., 2018). These results, together with the expression pattern results, indicated homologues of CRF4 in different species tend to have different expression patterns and diverge functionally.

Recent studies in *Arabidopsis* have shown that CRFs are not only responsive to the cytokinin, but also participate in the regulation of auxin transport (Schlereth et al., 2010; Ckurshumova et al., 2014; Simaskova et al., 2015; Wu et al., 2015; Cucinotta et al., 2016; Waidmann et al., 2019). CRF2 and CRF6 could bind to the promoters of auxin efflux carriers *PIN1* and *PIN7* and suppress their expression level (Simaskova et al., 2015). *PIN7* was upregulated in *crf6* mutants, and auxin content in the root tips of *crf3crf6* double mutants was increased compared with wild type (Simaskova et al., 2015). In addition, the loss of *CRF2* and *CRF3* led to changes in lateral root gravity (Waidmann et al., 2019). In this study, *GmCRF4a* may also be involved in the auxin pathway. The auxin content in mutant *Gmcrf4a-1* and co-silencing line (*CS*) was significantly reduced, while significantly increased in the *OX* lines (Figure 5A). Auxin governs the form and shape of the plant body by stimulating cell elongation, which might explain the cell elongation and plant height phenotype of *Gmcrf4a-1*, *CS and OX* lines (Velasquez et al., 2016). In order to clarify the source of auxin, expression of the key genes in auxin transport and synthesis pathways were detected. The results indicated that the auxin transporter *GmPINs* exhibited no difference in *Gmcrf4a-1*, *CS and OX* lines, while the auxin synthesis gene *GmYUCs* were significantly upregulated in *OX* lines and downregulated in *Gmcrf4a-1* and *CS lines* (Supplementary Figure S7; Figures 5B–D). *GmIAA14a* and *GmSAUR14a*, the downstream genes of auxin signaling transduction pathway, were also examined and the results showed opposite expression pattern in *GmCRF4a* mutants and overexpression lines. Overall, although the protein structure of *GmCRF4a* is more homologous to *Arabidopsis CRF4*, its expression profile is different, and its function is closer to *CRF2*, *CRF3* and *CRF6*. Different from *CRF2*, *CRF3* and *CRF6*, *GmCRF4a* did not affect auxin transport, but may affected auxin content by increasing auxin key synthase. Therefore, the present findings provide a new possible pathway for CRF to regulate plant height and enrich the functional cognition of CRFs.

In addition, the diffident phenotypes of *CS* and *OX1* in plant height and growth period under natural field condition provides two possible scenarios for their breeding applications. *CS* plants had a dwarf compact plant architecture and short growth period which make it possible to be introduced from low latitude to high latitude, and the yield may be increased through intercropping and reasonable dense planting. *OX1* displayed higher plant height, increased pod setting height, more nodes, and a longer growth period, so it was recommended to be introduced from high latitude to low latitude to achieve the possibly purpose of improving late-maturing yield. Taken together, our study provides an excellent candidate gene *GmCRF4a* and corresponding plant materials for genetic breeding to improve soybean architecture, which would facilitate future molecular breeding practice.

## Data availability statement

The datasets presented in this study can be found in online repositories. The names of the repository/repositories and accession number(s) can be found in the article/Supplementary material.

## Author contributions

TaZ, TuZ, and BL designed the studies and wrote the manuscript. ZX conducted the bioinformatic analyses. ZX, RW, KK, NB, AA, JL, and HL performed the experiments. All authors contributed to the article and approved the submitted version.

## Funding

This research was funded by the National Natural Science Foundation of China (32072091), the National Natural Science Foundation of China (32171965), Science and Technology Innovation Team of Soybean Modern Seed Industry in Hebei (21326313D), the Jiangsu Seed Industry Revitalization Project [JBGS(2021)014], and the Jiangsu Collaborative Innovation Centre for Modern Crop Production (JCIC-MCP) Program.

## Conflict of interest

The authors declare that the research was conducted in the absence of any commercial or financial relationships that could be construed as a potential conflict of interest.

## Publisher’s note

All claims expressed in this article are solely those of the authors and do not necessarily represent those of their affiliated organizations, or those of the publisher, the editors and the reviewers. Any product that may be evaluated in this article, or claim that may be made by its manufacturer, is not guaranteed or endorsed by the publisher.

## Footnotes

^1^https://phytozome-next.jgi.doe.gov/, accessed on January 7, 2022.

^2^https://venanciogroup.uenf.br/cgi-bin/gmax_atlas/index.cgi, accessed on January 5, 2022.

^3^http://crispr.hzau.edu.cn/CRISPR2/, accessed on January 5, 2022.

^4^ https://venanciogroup.uenf.br/cgi-bin/gmax_atlas/index.cgi
