# Supplementary material for: An APETALA2/ethylene responsive factor transcription factor GmCRF4a regulates plant height and auxin biosynthesis in soybean
Source: Front Plant Sci. 2022 Sep 6;13:983650. doi: 10.3389/fpls.2022.983650 (PMC9485679; doi:10.3389/fpls.2022.983650)
Supplement: Supplementary file 4 [file Data_Sheet_1.DOCX]

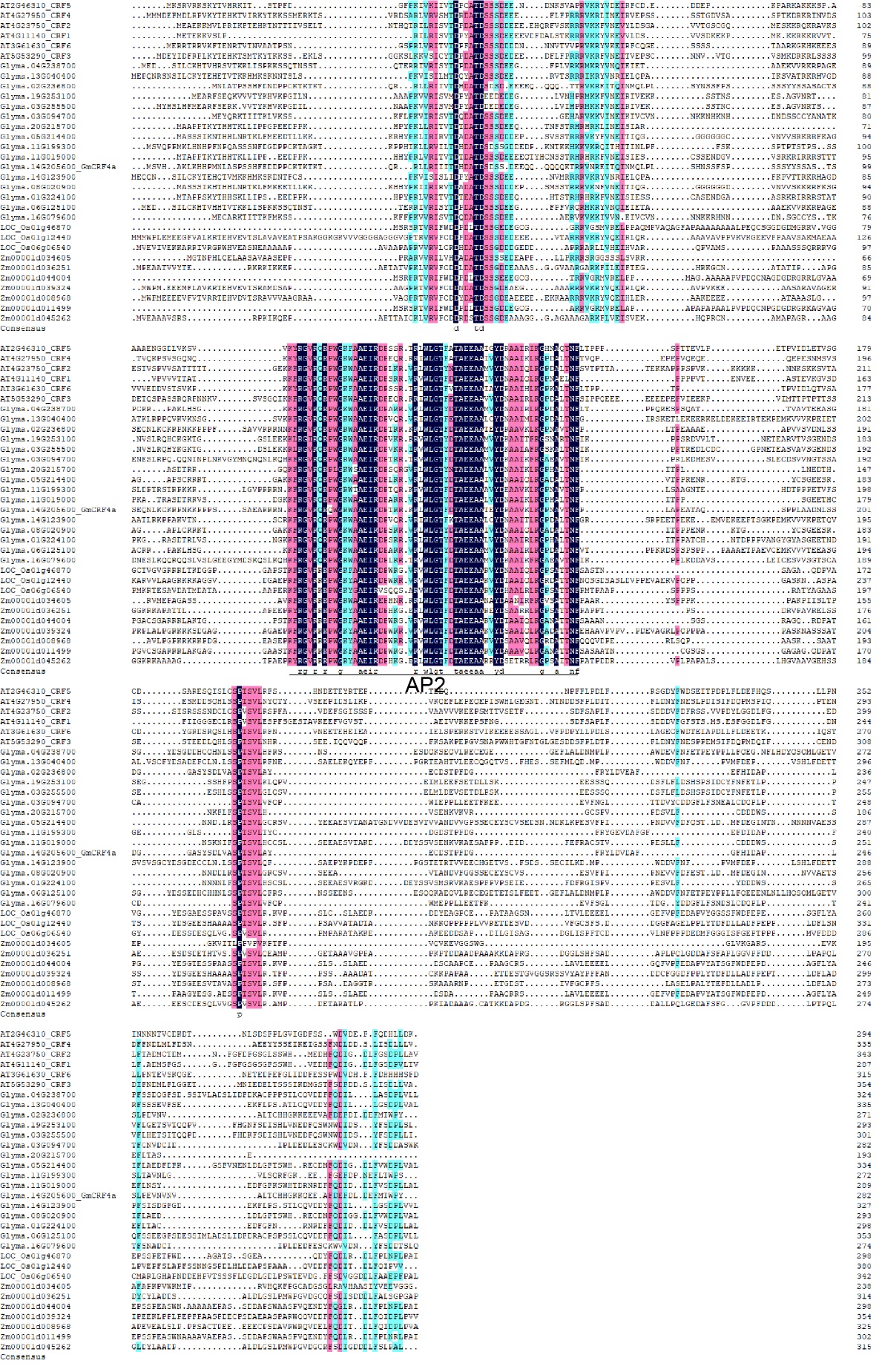


**Supplemental Figure 1.** Protein sequence and domain analysis of CRF4 homologous genes in *Arabidopsis*, Soybean, maize and rice.


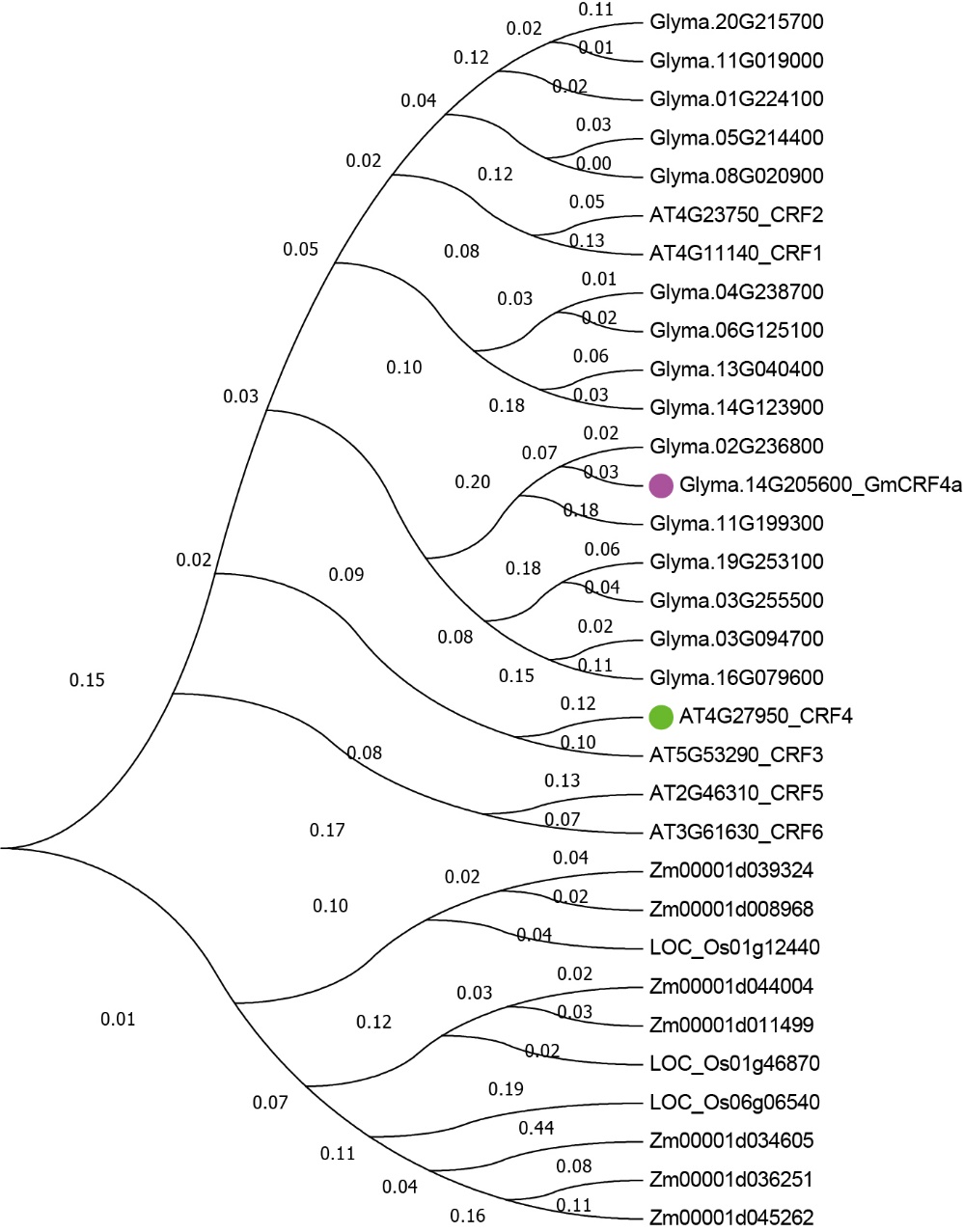


**Supplemental Figure 2.** Molecular phylogenetic analysis of CRF4 in Arabidopsis, soybean, maize and rice by Maximum Likelihood method. The green circle represents the CRF4 gene and the red circle represents the GmCRF4a gene. The numbers on the branches indicate the length of the branches.


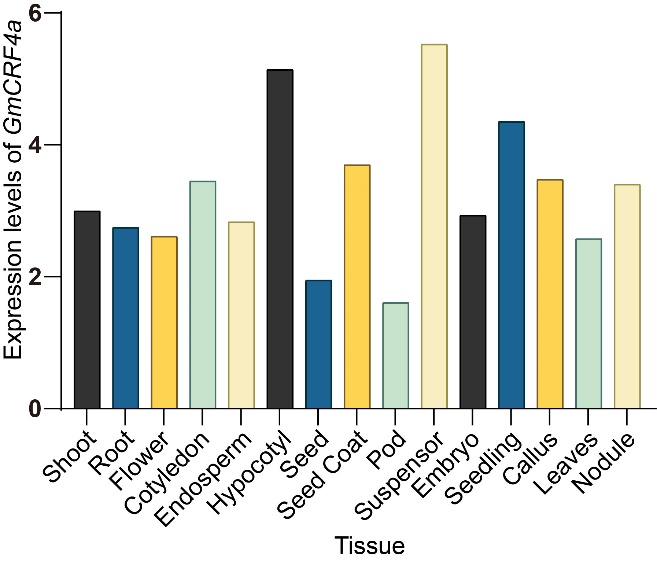


**Supplemental Figure 3.** *GmCRF4a* expression levels in various tissues from the published Soybean Expression Atlas.


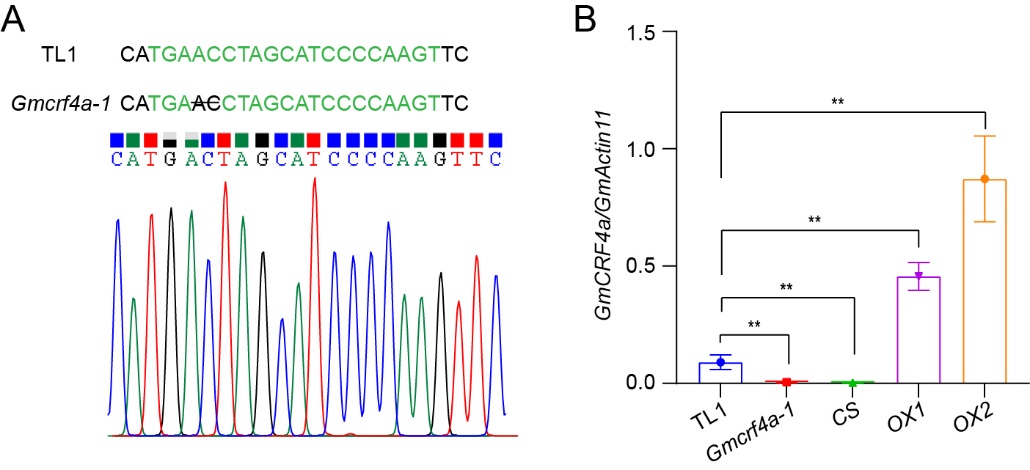


**Supplemental Figure 4.** The *Gmcrf4a-1* line sequencing and the expression level of *GmCRF4a* in indicated lines. (**A**), The base change and the sequencing data of *Gmcrf4a-1*, the black line within the target site, denotes nucleotide deletion. (**B**), Relative expression of *GmCRF4a* in different individuals. *GmActin11* as an internal control. The asterisk indicates a statistically significant difference from the wild type by a two-sided *t*-test (** *P* < 0.01). Data are displayed as means ± s.d. (*n* = 3).


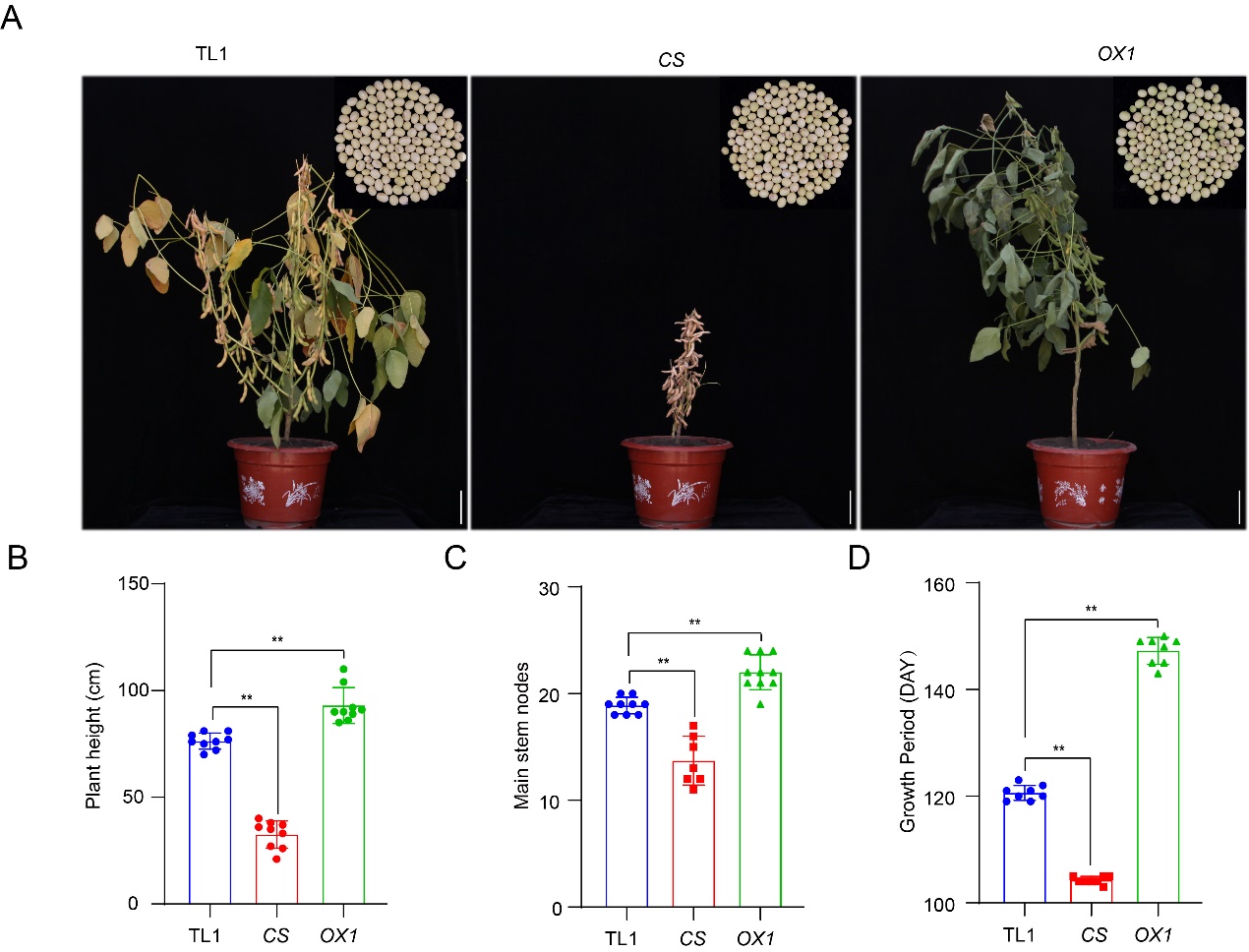


**Supplemental Figure 5.** Field phenotypes and statistics data of TL1, *CS*, and *OX1*. (**A**) Field phenotypes of TL1, *CS*, and *OX1*. The upper right corner indicated soybean seeds in the R8 stage. The number of seeds in the diagram did not represent the total number of grains per plant. Scale bars = 10 cm. (**B-D**) Comparison of plant height (**B**), main stem nodes (**C**), and growth period (**D**) of TL1, *CS*, and *OX1*. The asterisk indicates a statistically significant difference from the wild type by a two-sided *t*-test (** *P* < 0.01). Values are means ± s.d. (*n* > 8).


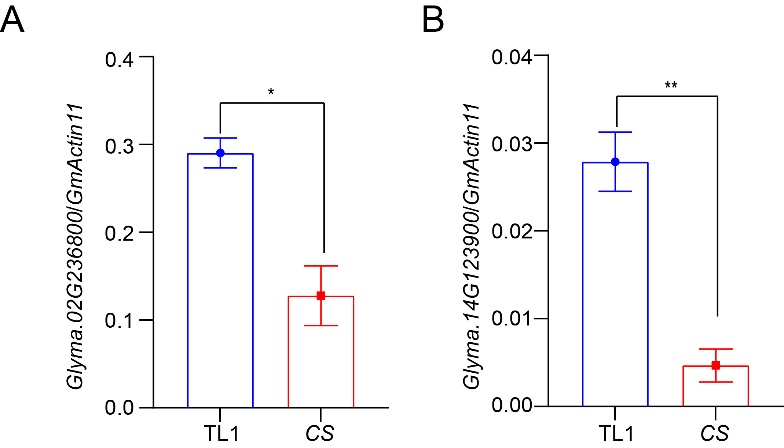


**Supplemental Figure 6.** Relative expression of *GmCRF4a* homologous gene *Glyma.02G236800* (A) and *Glyma.14G123900* (B) in TL1 and *CS*. *GmActin11* as an internal control. The asterisk indicates a statistically significant difference from the wild type by a two-sided *t*-test (* *P* < 0.05, ** *P* < 0.01). Data are displayed as means ± s.d. (*n* = 3).


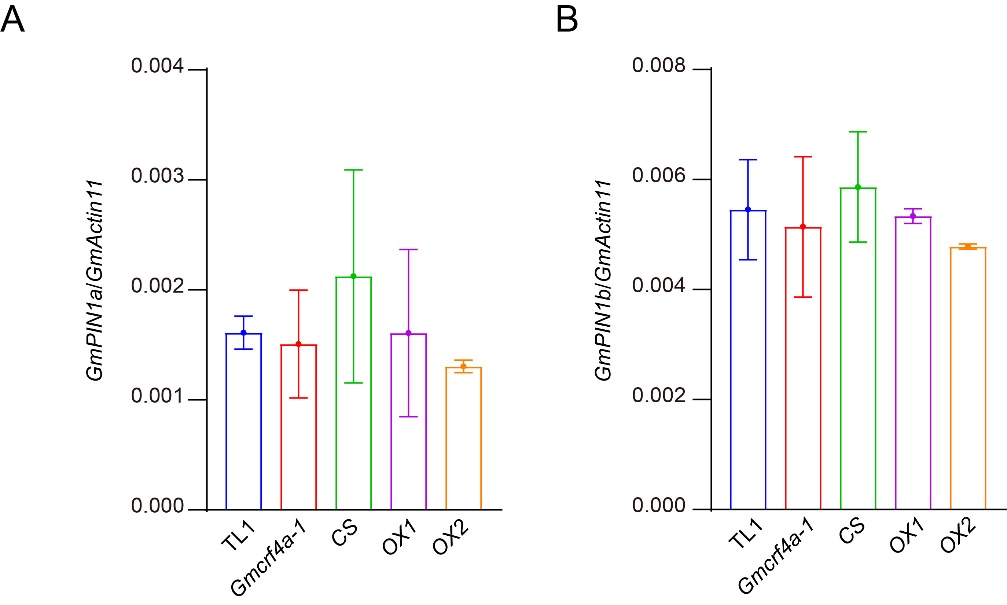


**Supplemental Figure 7.** Relative expression of *GmPIN1a* and *GmPIN1b* in different individuals. *GmActin11* as an internal control. Data are displayed as means ± s.d. (*n* = 3).
